# Systematic profiling of nudivirus-like genes reveals conserved and differentiated roles in a domesticated endogenous virus

**DOI:** 10.1371/journal.ppat.1014492

**Published:** 2026-08-05

**Authors:** Yuenan Zhou, Kaijie Ding, Sijie Zhang, Xin Yang, Zhiwei Wu, Jiachen Zhu, Xiqian Ye, Jianhua Huang, Xuexin Chen, Zhizhi Wang

**Affiliations:** 1 State Key Lab of Rice Biology, Ministry of Agriculture and Rural Affairs Key Lab of Molecular Biology of Crop Pathogens and Insects, and Zhejiang Provincial Key Laboratory of Biology of Crop Pathogens and Insects, Zhejiang University, Hangzhou, China; 2 Institute of Insect Sciences, College of Agriculture and Biotechnology, Zhejiang University, Hangzhou, China; 3 College of Advanced Agricultural Sciences, Zhejiang A&F University, Hangzhou, China; Fred Hutchinson Cancer Center, UNITED STATES OF AMERICA

## Abstract

Cotesia vestalis bracovirus (CvBV) is a type of domesticated endogenous virus (DEV) derived from ancestral nudiviruses that is integrated into the genome of the parasitoid wasp *Cotesia vestalis*. The CvBV proviral genome is composed of two distinct components: one encoding genes associated with virion morphogenesis and assembly, and the other harboring virulence genes that are excised, circularized, and packaged into virions. CvBV replication and particle assembly occur exclusively in the ovaries of female wasps. While prior studies have largely focused on the function of virulence genes during parasitization, the molecular mechanisms underlying CvBV replication and assembly remain poorly understood. Here, we identified 71 nudivirus-like genes in the *C. vestalis* genome through integrated transcriptomic and proteomic analyses. Using gene silencing and microscopy-based imaging approaches, we functionally characterized 24 key genes involved in DNA replication (*helicase*, *integrase-1*, and *integrase-2*), transcriptional regulation (*p47*, *lef-5*, and *lef-9*), capsid formation (*vp39*, *PmV*, *HzNVorf9–1*, *HzNVorf9–2*, *HzNVorf106*, *38k*, *27b*, and *K425_459*), envelope formation (*11k*, *17a-1*, *35a-1*, *35a-2*, and *K425_461*), virion assembly (*vlf-1*, *HzNVorf140–1*, and *HzNVorf140–2*), and viral infectivity (*pif-0* and *vp91*). Although the functions of most nudivirus-like genes are generally conserved among baculoviruses, nudiviruses, and bracoviruses, *lef-5*, *K425_459*, *11k*, and *vp91* appear to have undergone functional divergence relative to their homologs in baculoviruses, nudiviruses, and Microplitis demolitor bracovirus, highlighting lineage-specific adaptations in CvBV. Collectively, our work provides a molecular framework for understanding CvBV assembly and serves as a valuable resource for investigating bracovirus evolution.

## Author summary

Parasitic wasps have achieved an extraordinary evolutionary innovation by domesticating ancient viruses and turning them into essential biological tools. In the wasp *Cotesia vestalis*, a domesticated virus is permanently embedded in the wasp genome and produces viral particles that help the wasp overcome the immune defenses of its insect hosts. While the role of these viral particles during parasitism has been studied for years, how they are actually built inside the wasp has remained largely unknown. In this study, we set out to uncover the genetic machinery that allows this virus to replicate and assemble. By combining large-scale gene expression and protein analyses with targeted gene silencing and imaging, we identified dozens of virus-derived genes that work together to copy viral DNA, control gene activity, construct viral shells and membranes, assemble complete particles, and ensure their infectivity. We also found that several of these genes have evolved new functions that differ from their counterparts in related viruses, revealing how this domesticated virus has been fine-tuned to its wasp host. Together, our work provides a comprehensive and accessible framework for understanding how domesticated viruses are produced and offers new insights into the long-term evolution of virus–host partnerships.

## Introduction

Some parasitic wasps have accomplished a remarkable feat of evolution: they have tamed wild viruses, known as Domesticated Endogenous Viruses (DEVs), forging an enduring alliance that is now critical to their survival [1]. Two types of DEVs, bracoviruses (BVs) and ichnoviruses (IVs), are associated with the two largest parasitoid wasp families, Braconidae and Ichneumonidae (Insecta: Hymenoptera), respectively [1–3]. These two subgroups have independent origins: BVs derive from an ancient integration of a nudiviral ancestor into the genome of a braconid wasp lineage, whereas IVs originate from the integration of a different, yet unidentified, viral lineage into that of ichneumonid wasps [4–8]. The assembly and replication of both DEVs occur exclusively in the specialized ovarian calyx cells of pupal and adult female wasps. Mature BV virions are released into the calyx lumen through cell lysis, forming the calyx fluid, whereas IVs are released by budding from the plasma membrane [3,9–11]. Most DEV-carrying wasps parasitize lepidopteran larvae, and the associated DEVs function as mutualists to suppress host immunity and ensure the successful development of wasp offspring [12–15].

BVs contain one or more capsids, each of which encapsidates multiple circular, double-stranded DNA (dsDNA) segments (190–600 kb) encoding diverse virulence-associated genes [3]. The capsids are further enclosed by a single lipid bilayer envelope [16]. In bracoviruses, the BV genome comprises two distinct sets of integrated DNA segments: structural-related genes responsible for viral replication and assembly, and virulence genes packaged into virions, injected into the lepidopteran host hemocoel [12–15]. The structural genes are homologous to the core genes of two arthropod-infecting lineages of dsDNA viruses: nudiviruses and their sister group, baculoviruses [4]. In nudiviruses or baculoviruses, the conserved core genes function in viral DNA replication (e.g., *dnapol* and *helicase*), transcription (e.g., *lef-4*, *lef-8*, *lef-9*, and *p47* as subunits of the viral RNA polymerase; *lef-5* as a transcription initiation factor), DNA packaging, virion production and assembly (e.g., *38k*, *p33*, *p6.9*, *vlf-1*, and *vp39*), infectivity (e.g., *pif-0* to *pif-6*, and *pif-8*), and other processes (e.g., *Ac81*) [17–21]. Although BVs have retained many structural components and replication-associated genes from their nudiviral ancestor [22,23], they exhibit distinct characteristics, including avoiding infection of the host wasp and thus not provoking immune responses, possessing specific replication sites, and participating in the wasp gene regulatory network [12,22,24,25]. It is unknown how these nudivirus-like genes have been adapted to support the association with parasitoid wasps. While some of these genes have been experimentally validated in the braconid wasp *Microplitis demolitor* [26–28], recent work also used functional genomics and knockdown to characterize a domesticated nudivirus in *Venturia canescens* [29]. The majority remain uncharacterized, highlighting a critical gap in our understanding of their adaptive evolution.

Cotesia vestalis bracovirus (CvBV) is a DEV associated with the parasitoid wasp *Cotesia vestalis* (Hymenoptera: Braconidae), a larval endoparasitoid of the diamondback moth (*Plutella xylostella*, Lepidoptera: Yponomeutidae), a major cruciferous pest [30]. Recent genome sequences of CvBV and *C. vestalis* have provided valuable resources for investigating viral replication and virion assembly in pupal ovaries [30,31]. However, the mechanisms underlying segment circularization and encapsidation, as well as the gene sets involved, are yet to be elucidated.

In this study, we investigated the molecular regulators orchestrating CvBV biogenesis, including viral production, maturation, and virion release. Building on our previous identification of 30 CvBV DNA segments [31], we quantified the abundance of circular CvBV segments across pupal and adult ovary stages and identified 71 nudivirus-like genes through integrated transcriptomic and proteomic analyses. To functionally characterize these candidate genes, we combined absolute quantitative PCR (qPCR), transmission electron microscopy (TEM), and RNA interference (RNAi) to assess their roles in DNA replication, transcriptional regulation, virion morphogenesis, and viral infectivity.

## Results

### De novo assembly of CvBV virions in the calyx cell nucleus of *C. vestalis*

**Fig 1:**
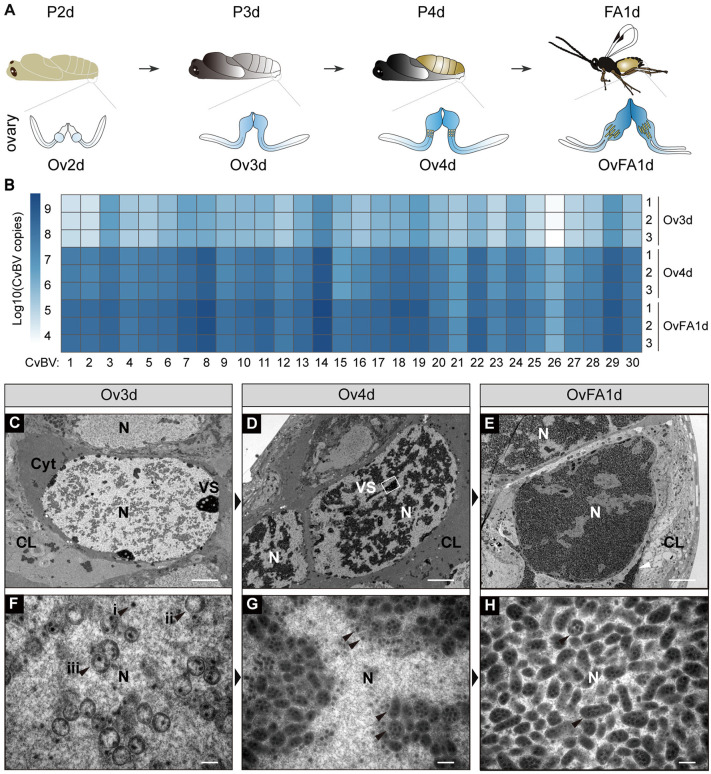
Production of CvBV virions in *C. vestalis* calyx cells during pupal development. **(A)** *C. vestalis* pupal-to-adult development timeline with corresponding ovarian images. Yellow regions indicate maturing or fully mature eggs at Ov4d and OvFA1d. P2d, 2-day-old pupa; P3d, 3-day-old pupa; P4d, 4-day-old pupa; FA1d, 1-day-old female adult; Ov, Ovary. **(B)** Heatmap showing the abundance of CvBV circles 1–30 across ovarian development from Ov3d to OvFA1d. Rows represent ovary developmental stages, with three biological replicates per stage (*n* = 3), and columns correspond to CvBV circles 1–30. Color intensity reflects log_10_-transformed CvBV copy numbers (raw CvBV copy numbers and corresponding raw and adjusted *p*-values are provided in S1 Table), with darker colors indicating higher abundance. Statistical significance was assessed using two-sided *t*-tests with Benjamini–Hochberg correction. **(C-E)** TEM overview of virion morphogenesis at Ov3d, Ov4d, and OvFA1d, respectively. The white rectangle in **(D)** marks the virogenic stroma, and the magnified view is shown in S1M Fig. The white arrow in **(E)** indicates mature virions present in the calyx lumen. **(F-H)** Enlarged views showing virion assembly within a calyx cell nucleus at Ov3d, Ov4d, and OvFA1d, respectively. In **(F)**, black arrows indicate three representative structures: **(i)** an unenveloped nucleocapsid (a small electron-dense dot); **(ii)** an empty envelope without a nucleocapsid (a rounded membrane structure); and **(iii)** an assembling virion containing a nucleocapsid enclosed by a unit-membrane envelope (an electron-dense dot within a membrane-bound circle). Black arrows in **(G)** indicate unenveloped nucleocapsids (small electron-dense dots) and mature virions containing multiple nucleocapsids within a single envelope (multiple dots inside one circle). Black arrows in **(H)** indicate mature virions. N, nucleus. Cyt., cytoplasm. VS, virogenic stroma. CL, calyx lumen. Scale bars: **(C–E)**, 5 μm; **(F–H)**, 0.2 μm.

BV replication begins with amplification of the proviral genome, followed by virion assembly within calyx cell nuclei [11,23]. In *C. vestalis*, pupal development lasts ~4.5 days [32] and is accompanied by ovarian development and CvBV virion assembly (Fig 1A and S1A-I Fig). We previously identified 30 dsDNA circles in the CvBV genome and characterized wasp integration motifs [31]. Absolute qPCR targeting the integration sites showed that the abundance of circularized CvBV DNAs was zero at Ov2d (S1 Table), first appeared at Ov3d, and increased sharply by Ov4d, peaking at OvFA1d (Fig 1B; raw CvBV copy numbers and *p*-values are shown in S1 Table). The relative abundance of individual CvBV circles appears to be established as early as the Ov3d stage, with circles of high abundance remaining dominant and low-abundance circles staying consistently low throughout ovarian development. For example, CvBV circle 14 shows the highest abundance at Ov3d and continues to be the most abundant circle during both Ov4d and OvFA1d. In addition, the other five circles in the top-six group (8, 29, 7, 18, and 19) exhibit a similar pattern. DAPI staining and TEM images showed progressive enlargement of calyx cells and nuclei from Ov3d to OvFA1d (S1J-L Fig), with the cytoplasm still visible at Ov3d but completely absent at the late stages Ov4d and OvFA1d (Fig 1C-E). TEM image showed that CvBV virion formation was first observed at Ov3d, where capsids and envelopes began to co-assemble (Fig 1F). At Ov4d, some assembled virions are visible, consisting of multiple nucleocapsids enclosed by a single-layer envelope (Fig 1G), while many unassembled nucleocapsids are present at the periphery of the virogenic stroma, awaiting assembly (S1M Fig). At OvFA1d, the calyx cell nucleus was filled with mature virions (Fig 1H). As calyx cells lysed, these mature virions mixed with the wasp egg (S1N and S1O Fig), and were then injected together into the host larvae during parasitization. The concordance between qPCR and TEM results supports the proposed temporal sequence of CvBV replication and virion morphogenesis.

### Dynamic expression of CvBV nudivirus-like genes during pupal ovary development of *C. vestalis*

To investigate gene expression programs underlying CvBV virion formation, we performed RNA-seq on ovaries from five developmental stages using the Illumina HiSeq 2500 platform. Approximately 707 million raw reads were generated from all samples, with clean reads exhibiting mapping ratios ranging from 94% to 97% (S2 Table). These high-quality RNA-seq data enabled us to explore gene expression dynamics across developmental stages. We next conducted a genome-wide analysis of gene expression levels in *C. vestalis* based on our newly assembled genome and annotation (GenBank accession No.: GCA_054085095.1). K-means clustering (*k* = 8) identified eight clusters with distinct temporal expression dynamics across the five ovarian developmental stages in *C. vestalis* (S2A Fig).

We first identified 51 nudivirus-like genes from the *C. vestalis* genome annotation based on homology to nudivirus-like genes from *C. congregata*. We then performed an additional BLASTP search using RNA-seq de novo–assembled transcripts to recover genes missing from the genome annotation, resulting in the identification of 20 additional nudivirus-like genes. Together, this integrated genome- and transcriptome-based approach resulted in a total of 71 nudivirus-like genes, ensuring a more complete identification of the nudiviral gene set (S3 Table). Notably, all are single-exon genes and share 66–99% amino acid identity with *C. congregata* homologs [22], supporting the accuracy of our annotation. To better understand the genomic context of these nudivirus-like genes, we examined their chromosomal distribution and organization. These genes are distributed across nine chromosomes and organized into three major clusters: cluster 1 on chromosome 7 (23 genes, ~ 82 kb), cluster 2 on chromosome 10 (6 genes, ~ 11 kb), and cluster 3 on chromosome 2 (3 genes, ~ 3.3 kb), with clusters 2 and 3 composed of *odv-e66* family genes (S2B Fig). Interestingly, most clusters except cluster 6 identified in *C. vestalis* were also present in *C. congregata* [22], where they similarly exhibit clustered genomic organization.

**Fig 2:**
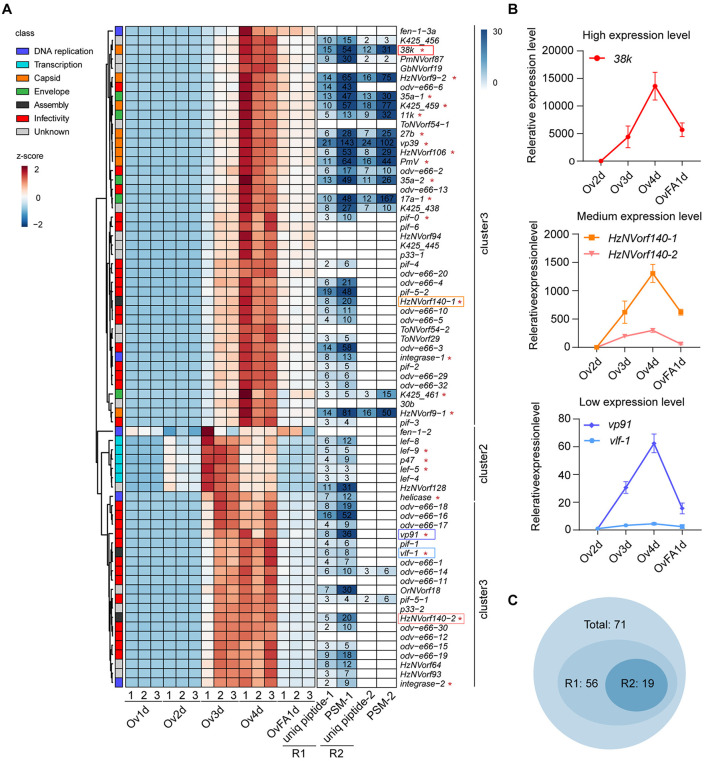
Conserved nudivirus-like genes identified in *C. vestalis.* **(A)** Left panel: Heatmap showing scaled expression levels (z-score) of 71 nudivirus-like genes in *C. vestalis* ovaries across different developmental stages. Expression values were calculated as TPM from three biological replicates for each developmental stage. Differential expression analysis was performed using DESeq2, with Ov1d as the reference condition. Log_2_ fold changes and adjusted *p*-values are provided in S4 Table. Blue and red indicate lower and higher expression, respectively. Colored squares along the clustering tree represent predicted viral functions based on experimental validation and baculovirus homology. Right panel: Heatmap depicting mass spectrometry-based peptide detection for the 71 genes. Numbers within the boxes represent unique peptides and PSMs identified by mass spectrometry (only values ≥ 2 are shown). Darker blue shades indicate higher peptide counts, with values above 30 shown at the maximum blue intensity. Box colors indicate genes with high, medium, or low expression levels, consistent with validation in **(B)**. The 24 genes marked with red asterisks were subjected to functional validation by RNAi in this study. K-means analysis (see S2A Fig) grouped the genes into two clusters, with cluster labels shown to the right of each gene name. The gene *fen-1-2* was excluded from the K-means clustering due to its extremely low expression. **(B)** qPCR analysis (*n* = 3 biological replicates) of five nudivirus-like genes in *C. vestalis* shows expression patterns consistent with those observed in the heatmap **(A)**. **(C)** Mass spectrometry-based proteomic analyses of calyx fluid (R1) and purified CvBV virions (R2). R1 identified 56 proteins and R2 19 proteins, each supported by ≥ 2 unique peptides.

RNA-seq analysis revealed dynamic expression of all 71 nudivirus-like genes in *C. vestalis* ovaries during pupal-to-adult development (S4 Table). Some genes showed peak expression at Ov3d and declined thereafter (early-expressed; corresponding to K-means cluster 2), while others peaked at Ov4d (late-expressed; corresponding to K-means cluster 3) (Fig 2A). Notably, functional predictions based on homology suggest that early-expressed genes were mainly associated with DNA replication and transcriptional regulation, whereas late-expressed genes may contribute to structural components and virion assembly factors. qPCR validation of five representative genes (*38k*, *HzNVorf140–1*, *HzNVorf140–2*, *vlf-1*, and *vp91*) confirmed the transcriptome patterns (Fig 2B). These results provide the first comprehensive catalog of nudivirus-like genes in *C. vestalis* and reveal their temporally coordinated expression during ovary development.

### CvBV virions contain conserved nudivirus-like proteins

To distinguish structural proteins of CvBV virions from other proteins, we performed proteomic analyses on both calyx fluid (R1) and purified CvBV virions (R2, including two technical replicates: R2-1 and R2-2), which allowed us to compare their protein compositions (S3A Fig). To identify nudivirus-like proteins, mass spectra were searched against a custom protein database translated from the *C. vestalis* ovary transcriptome (S5 and S6 Tables). After summing the two technical replicates of R2 for both unique peptides and peptide-spectrum matches (PSMs), we assessed their consistency with R1. This analysis showed strong correlations (*r* = 0.79 for unique peptides, *r* = 0.60 for PSMs), and FPKM (Fragments Per Kilobase of transcript per Million mapped reads) showed a reasonable correlation with proteomic measurements in both samples (R1: *r* = 0.49–0.65; R2: *r* = 0.61–0.75; S3B Fig).

We hypothesized that sample R1 likely contains nearly all CvBV nudivirus-like proteins and partial wasp proteins, while sample R2 is expected to be enriched for structural components of CvBV virions. Of the 71 nudivirus-like proteins, 56 highly expressed proteins in R1 were identified with at least two unique peptides, while 19 were identified in R2 with at least two unique peptides (Fig 2C; S5 and S6 Tables). Proteins in R2 were predominantly highly expressed capsid or envelope components, e.g., capsid protein *vp39* (21 unique peptides in R1, 24 in R2), the most highly expressed viral transcript (Fig 2A; S4 Table). As expected, several early-expressed genes with homology to nudivirus genes were exclusively identified in sample R1, including *helicase* (7 unique peptides), *p47* (4), *lef-8* (6), *lef-9* (5), *lef-4* (3), and *lef-5* (3). Taken together, our proteomic analysis allows us to tentatively distinguish major structural proteins of CvBV from auxiliary factors involved in viral replication and virion maturation.

### CvBV nudivirus-like genes are efficiently knocked down by RNAi

To functionally verify CvBV nudivirus-like genes, we selected 24 highly expressed genes for RNAi knockdown based on transcriptomic and proteomic data (Fig 2A and S4A Fig). These genes were grouped into four functional categories:

(1) DNA replication-related: *helicase*, *integrase-1*, and *integrase-2*;
(2) transcription-related: *p47*, *lef-5*, and *lef-9*;
(3) a functionally validated homolog or paralog in baculoviruses, nudiviruses, or other bracoviruses—many of which are implicated in virion structure or assembly—although whether these genes are conserved functionally in CvBV remains unclear: *vp39*, *38k*, *HzNVorf9–1*, *HzNVorf9–2*, *HzNVorf106*, *27b*, *K425_459*, *11k*, *17a-1*, *35a-1*, *35a-2*, *vlf-1*, *HzNVorf140–1*, *HzNVorf140–2*, *pif-0*, and *vp91*;
(4) highly expressed genes of unknown function: *PmV* and *K425_461*.

Double-stranded RNAs (dsRNAs) were injected into one-day-old female pupae to optimize gene silencing. Using the late-expressed *38k* as a test case, we found that 200–400 ng dsRNA achieved >99% knockdown as measured by qPCR (S4B Fig). RNAi of all 24 genes yielded knockdown efficiencies of 74–98% (S4B Fig). To assess potential off-target effects of RNAi, we first performed in silico analyses of sequence similarity among CvBV nudivirus-like genes using BLASTN, identifying a limited number of short regions (≥ 21 nt) of potential homology between dsRNA target regions and non-target genes (S4C Fig). These included shared homologous fragments between *HzNVorf9–1*/*HzNVorf9–2*, *vp91*, and *PmNVorf87*, as well as between *HzNVorf106* and *helicase*. To experimentally evaluate RNAi specificity, we further quantified the expression of target genes, predicted off-target genes, and two *C. vestalis* housekeeping genes by qPCR following dsRNA treatment. Except for two *C. vestalis* genes (*dnapolδ* and *ef1α*), all primers were designed to span the predicted off-target regions. qPCR analyses showed no significant off-target effects were detected under the experimental conditions used (S4D and S4E Fig). Together, these results confirm efficient and specific RNAi-mediated gene silencing, supporting subsequent phenotypic analyses.

### Early-expressed genes coordinate CvBV replication and late-expressed gene transcription

**Fig 3:**
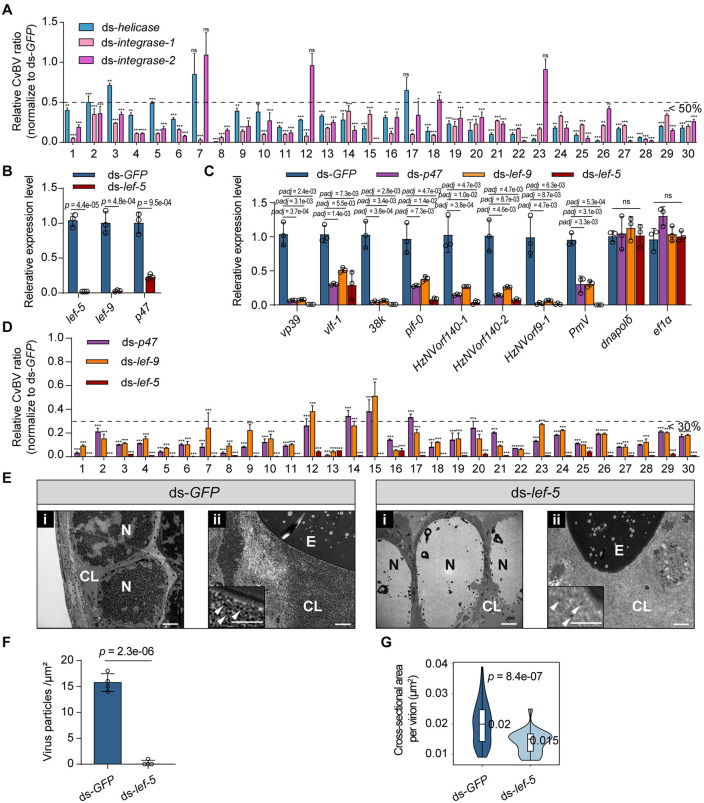
RNAi of early-expressed genes reduces CvBV abundance and downregulates late-expressed gene expression. **(A)** qPCR analysis showing relative abundance of CvBV circles 1-30 in FA1d female wasps pretreated with ds-*helicase*, ds-*integrase-1*, or ds-*integrase-2*, compared to ds-*GFP*-injected controls (*n* = 3 biological replicates). The y-axis represents the relative CvBV ratio for each sample, calculated as the CvBV copy number in each sample divided by the mean copy number of the three ds-*GFP*-injected replicates (raw CvBV copy numbers and corresponding raw and adjusted *p*-values are provided in S7 Table). **(B)** qPCR analysis of *lef-5*, *lef-9*, and *p47* expression levels in FA1d female wasps pretreated with ds-*GFP* or ds-*lef-5* (*n* = 3 biological replicates). **(C)** qPCR analysis of relative expression levels of eight randomly selected late-expressed viral genes and wasp genes in FA1d female wasps pretreated with ds-*GFP*, ds-*p47*, ds-*lef-9*, or ds-*lef-5* (*n* = 3 biological replicates). Wasp genes *dnapolδ* and *ef1α* were used as negative controls. Adjusted *p*-values (*padj*) are shown. **(D)** qPCR analysis showing relative abundance of CvBV circles 1-30 in FA1d female wasps pretreated with ds-*p47*, ds-*lef-9*, or ds-*lef-5*, compared to ds-*GFP*-injected controls (*n* = 3 biological replicates). The y-axis represents the ratio of CvBV copy numbers relative to ds-*GFP* controls, as defined in **(A)** (raw CvBV copy numbers and corresponding raw and adjusted *p*-values are provided in S8 Table). **(E)** TEM images of virion morphogenesis in FA1d female wasps pretreated with ds-*GFP* or ds-*lef-5*. White arrows with black outlines indicate mature virions in the lower-left insets of ds-*GFP* **(ii)** and ds-*lef-5* **(ii)**. **(F)** Virion density (number per μm²) in the calyx lumen of FA1d female wasps pretreated with ds-*GFP* or ds-*lef-5*, as quantified from TEM images (*n* = 4 biological replicates, measured from a 1 μm² central region selected in each of four independent images). **(G)** Cross-sectional area (μm²) of individual virions in the calyx lumen of FA1d female wasps pretreated with ds-*GFP* or ds-*lef-5*, as quantified from TEM images. A total of *n* = 50 randomly selected, non-overlapping virions without obvious aggregation were measured in each group. Median values are indicated on the plot. N, nucleus; E, wasp egg; CL, calyx lumen. Scale bar: 5 μm. **(A**, **C**, and **D)** Statistical significance was assessed using two-sided *t*-tests with Benjamini–Hochberg correction. *padj< 0.05; ** *padj*< 0.01; *** *padj*< 0.001; ns, not significant. **(B**, **F**, and **G)** Statistical significance was assessed using two-sided *t*-tests.

*P143* helicase binds DNA in baculoviruses, and *integrase-1* is required for proviral DNA excision in *M. demolitor* [20,27]. In *C. vestalis*, early-expressed genes linked to DNA replication and transcriptional regulation (Fig 2A) were detected at high levels by LC-MS/MS, including *helicase* and *integrase-1/-2*. RNAi knockdown of ds-*helicase*, ds-*integrase-1*, and ds-*integrase-2* each led to a significant reduction (>50%) in the abundance of CvBV circles, as determined by the average remaining CvBV abundance ratio across three biological replicates (Fig 3A; raw CvBV copy numbers and *p*-values are shown in S7 Table). Specifically, ds-*helicase* and ds-*integrase-2* treatments reduced the abundance of 26 circles, while ds-*integrase-1* treatment decreased all 30 circles below 50%. Collectively, these results suggest that *helicase*, *integrase-1*, and *integrase-2* may play conserved roles and broadly influence the abundance of CvBV circles 1–30.

Other early-expressed genes include nudivirus-like transcriptional regulators *p47*, *lef-4*, *lef-9*, and *lef-5*. Given its homology to the baculovirus Autographa californica multiple nucleopolyhedrovirus (AcMNPV) and other systems, *lef-4* and *lef-9* act as RNA polymerase subunits, while *lef-5* initiates late-expressed gene transcription [21,27,33]. RNAi showed that ds-*lef-5* strongly suppressed the expression of two other early-expressed genes, *p47* and *lef-9*, and most late-expressed genes (*vp39*, *vlf-1*, *38k*, *pif-0*, *HzNVorf140–1*, *HzNVorf140–2*, *HzNVorf9–1*, and *PmV*) (Fig 3B and 3C). Similarly, ds-*p47* and ds-*lef-9* treatments also significantly reduced the expression of late-expressed genes, consistent with their roles in transcriptional regulation (Fig 3C). Given the putative role of late-expressed genes as structural components of CvBV virions (including but not limited to *vp39*) [27], we hypothesized that their reduced expression would result in decreased virion production. As expected, ds-*p47* and ds-*lef-9* treatments significantly reduced the abundance of most CvBV circles to below 30% (*p47*: 27 circles; *lef-9*: 28 circles), whereas ds-*lef-5* treatment decreased the abundance of all CvBV circles to below 6% (Fig 3D; raw CvBV copy numbers and *p*-values are shown in S8 Table). TEM further confirmed severe impairment of virion production after ds-*lef-5* injection (Fig 3E and 3F). Compared with ds-*GFP* controls, virions were rarely observed in either the calyx cell nuclei or the calyx lumen in ds-*lef-5*–treated wasps. Quantitative analysis of TEM cross-sectional areas further suggested that CvBV virions in ds-*lef-5*–treated wasps were noticeably smaller than those in the ds-*GFP* control group (Fig 3G). Together, these results suggest that these early-expressed genes play important roles in CvBV genome abundance and virion formation.

### Functional characterization of capsid and envelope genes in CvBV

In *M. demolitor*, the late-expressed genes are mainly associated with structural proteins and viral assembly-related components [23,27]. In *C. vestalis*, the number of genes encoding capsid and envelope proteins remains unclear. LC-MS/MS analysis identified *vp39* as the most abundant protein, with the highest number of unique peptides in both calyx fluid (R1: 21) and purified virions (R2: 24) (Fig 2A; S3 Table). Twelve additional proteins (*PmV*, *38k*, *HzNVorf9–1*, *HzNVorf9–2*, *HzNVorf106*, *27b*, *K425_459*, *11k*, *17a-1*, *35a-1*, *35a-2*, and *K425_461*) also showed high unique peptide counts and peptide-spectrum matches (PSM ≥ 15) in the R2 sample, supporting their roles as CvBV structural components, though their specific localization within the virion remains unclear.

**Fig 4:**
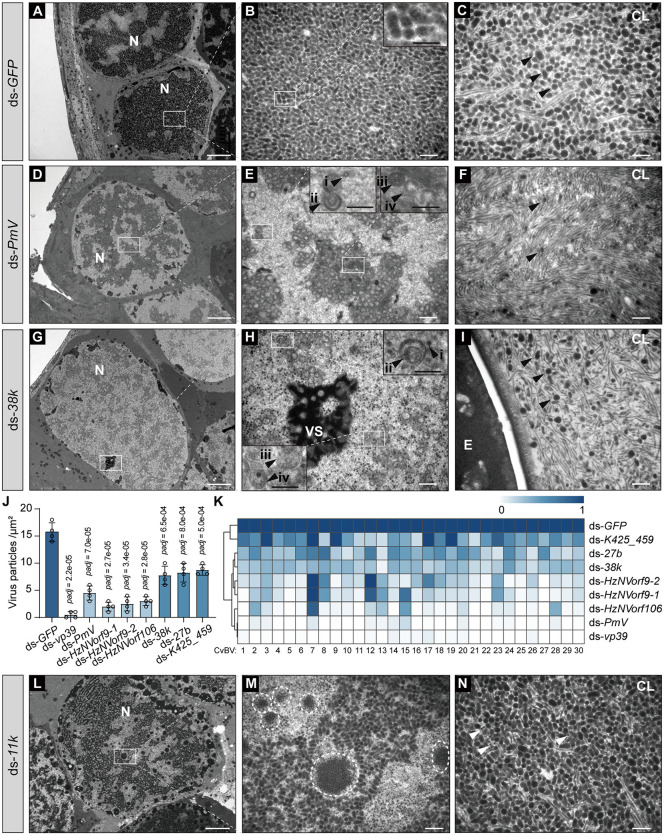
CvBV structural proteins are critical for virion integrity. **(A–I)** TEM images of virion morphogenesis in FA1d female wasps after RNAi knockdown of capsid genes. **(A, D, and G)** Overview of calyx cell nuclei in FA1d female wasps pretreated with ds-*GFP*, ds-*PmV*, or ds-*38k*. **(B, E, and H)** Magnified views of the boxed areas in **(A, D, and G)**, respectively. Insets (black boxes) show regions outlined by white boxes, illustrating the effects of RNAi on virion assembly. **(E)** Black arrows indicate four types of aberrantly assembled virions: **(i)** an incomplete nucleocapsid lacking an envelope (a small hollow circle); **(ii)** a single large, irregularly shaped nucleocapsid enclosed by an envelope (a large hollow circle containing a slightly smaller hollow circle); **(iii)** multiple incomplete nucleocapsids enclosed by an envelope (a large hollow circle containing several small hollow circles); and **(iv)** an empty envelope lacking nucleocapsids (a large hollow circle), which represents the most frequently observed phenotype. **(H)** Black arrows indicate four types of aberrantly assembled virions: **(i)** a nucleocapsid lacking an envelope (a small electron-dense dot; different from **(E)(i)**); **(ii)** a single large, irregularly shaped nucleocapsid enclosed by an envelope (a large hollow circle containing a slightly smaller hollow circle; same as **(E)(ii)**); **(iii)** a single small incomplete nucleocapsid enclosed by an envelope (a large hollow circle containing a small hollow circle; similar to **(E)(iii)**); **(iv)** a partially assembled virion containing only a nucleocapsid enclosed by a single-membrane envelope (an electron-dense dot within a membrane-bound circle; different from **(E)(iv)**). **(C, F, and I)** TEM images of the calyx lumen of FA1d female wasps pretreated with ds-*GFP*, ds-*PmV*, or ds-*38k*. Black arrows indicate mature virions. **(J)** Virion density (number per μm²) in the calyx lumen of FA1d female wasps pretreated with ds-*GFP*, ds-*vp39*, ds-*PmV*, ds-*HzNVorf9-1*, ds-*HzNVorf9-2*, ds*-HzNVorf106*, ds-*38k*, *ds-27b*, or ds*-K425_459*, as quantified from TEM images (*n* = 4 biological replicates, measured from a 1 μm² central region selected in each of four independent images). *padj* values are shown. **(K)** Heatmap showing CvBV relative abundance in ovaries of FA1d female wasps pretreated with nine dsRNAs shown in **(J)** (*n* = 3 biological replicates). In the heatmap, the color of each cell represents the mean ratio of CvBV copy numbers from three biological replicates relative to the ds-*GFP* controls (raw CvBV copy numbers and corresponding raw and adjusted *p*-values are provided in S9 Table). The x-axis represents individual CvBV circles (circles 1–30). Lighter colors indicate a greater reduction in CvBV abundance following knockdown. **(L–N)** TEM images of virion morphogenesis in FA1d female wasps after RNAi knockdown of envelope gene *11k*. **(L)** Overview of the calyx cell nuclei in FA1d female wasps pretreated with ds-*11k.* **(M)** Magnified view of the boxed area shown in **(L)**. White dashed circles highlight regions where multiple nucleocapsids, lacking an envelope, are aggregated together. **(N)** TEM image of calyx lumen in FA1d female wasps pretreated with ds-*11k.* White arrows indicate nucleocapsids not enclosed by an envelope. N, nucleus; VS, virogenic stroma; E, wasp egg; CL, calyx lumen. Scale bars: **(A, D, G, and L)**, 5 μm; **(B, C, E, F, H, I, M, and N)**, 0.5 μm; insets of **(B, E, and H)**, 0.25 μm. Statistical significance was assessed using two-sided *t*-tests with Benjamini–Hochberg correction.

Previous studies have shown that knockdown of capsid proteins disrupts bracovirus integrity and decreases viral DNA abundance [27,28]. For example, *vp39*, which encodes a nucleocapsid structural protein in baculoviruses, showed no apparent effect on envelope formation upon knockdown. However, TEM analysis revealed the presence of irregularly assembled virions, including enveloped particles containing aberrant or incomplete nucleocapsids, as well as empty envelopes completely lacking nucleocapsids, which accumulated and filled the nucleus. We hypothesize that knockdown of capsid genes in *C. vestalis* results in decreased CvBV abundance and aberrant virion formation, which may help distinguish capsid proteins from envelope proteins based on phenotypic differences observed. As expected, TEM analysis revealed severe structural defects in CvBV virions following *vp39* knockdown (S5A-C Fig). In the calyx cell nucleus, envelope formation appeared largely unaffected, with envelopes still being normally produced. However, these envelopes rarely contained nucleocapsids. No nucleocapsids were detected near the virogenic stroma, and correspondingly, few to no mature virions were observed in the calyx lumen. Similarly, knockdown of *PmV*, *HzNVorf9–1*, *HzNVorf9–2*, and *HzNVorf106* caused comparably severe phenotypes. Envelopes were still present but contained aberrantly assembled nucleocapsids, and the number of mature virions in the calyx lumen was markedly reduced compared with ds-*GFP* controls (Fig 4A-F and S5D-L Fig). In addition, knockdown of the other three genes (*38k*, *27b*, and *K425_459*) also affected viral capsid protein accumulation and nucleocapsid formation (Fig 4G-I and S5M-R Fig). However, depletion of the three genes was not sufficient to completely abolish nucleocapsid assembly, as a subset of nucleocapsids was still observed within the nucleus. Consistent with this partial defect, a small number of virions were still observed in the calyx lumen. Furthermore, all capsid gene knockdowns led to significantly reduced virion density in 1 μm² calyx lumen areas, significantly decreased virion cross-sectional area, and a marked global decrease in CvBV DNA abundance (Fig 4J, 4K, and S6 Fig). Specifically, injection of capsid genes dsRNA significantly reduced the average levels of CvBV to 1.9–55.5% of control levels, with *vp39* knockdown causing the most severe reduction (raw CvBV copy numbers and *p*-values are shown in S9 Table). Together, these results indicate that knockdown of capsid genes primarily disrupts nucleocapsid assembly and encapsidation rather than envelope biogenesis, leading to defective virion assembly and a pronounced reduction in CvBV abundance.

In addition to capsid genes, the number and functional roles of envelope genes in CvBV remain largely unexplored. The envelope primarily forms the outer membrane structure that surrounds nucleocapsids. Knockdown of envelope-associated genes may impair the efficiency of nucleocapsid envelopment and, in some cases, also affect viral assembly efficiency and/or the infectivity of nucleocapsids. A previous report showed that envelope gene *HzNVorf64* knockdown affects the abundance of a subset of Microplitis demolitor bracovirus (MdBV) circles, rather than causing a global reduction in viral circle abundance [26]. Therefore, we reasoned that CvBV envelope genes can be distinguished from capsid genes by combining TEM-based assessment of virion morphology with absolute qPCR quantification of CvBV circle abundance. Consistent with this expectation, knockdown of *11k* resulted in a pronounced accumulation of unenveloped nucleocapsids in the calyx cell nuclei from one-day-old female adults (Fig 4L and 4M). These nucleocapsids frequently appeared in clustered aggregates and were markedly more abundant than those observed in ds-*GFP* controls. The density of mature virions in the calyx lumen was markedly different from that observed in capsid gene knockdown groups, and showed no obvious difference compared with ds-*GFP* controls (Fig 4N). Furthermore, RNAi targeting four additional genes (*17a-1*, *35a-1*, *35a-2*, and *K425_461*) produced similar phenotypes in TEM images (S7A-L Fig). Nucleocapsid morphology was largely unchanged, but a small number of nucleocapsids clustered within the nucleus. Some unassembled nucleocapsids were also released into the calyx lumen following cell lysis. Based on TEM images of the calyx lumen, we quantified the number of mature virions and found no obvious difference compared with ds-*GFP* controls (S7M Fig). Quantification of virion cross-sectional areas further suggested that knockdown of *17a-1* and *35a-1*/*35a-2* moderately reduced virion size, whereas no obvious size reduction was observed following *11k* or *K425_461* knockdown (S7N Fig). Similarly, qPCR analysis of CvBV circles 1–30 showed that knockdown of any single gene did not cause a global change in CvBV circle abundance and that only a subset of circles exhibited moderate up- or down-regulation (S7O Fig; raw CvBV copy numbers and *p-*values are shown in S10 Table). Overall, the TEM phenotypes and qPCR results support a clear distinction between the CvBV envelope and capsid genes. Envelope genes appear to primarily affect nucleocapsid envelopment efficiency, without broadly altering nucleocapsid structure or viral circle abundance.

### Functional characterization of CvBV assembly-related genes

**Fig 5:**
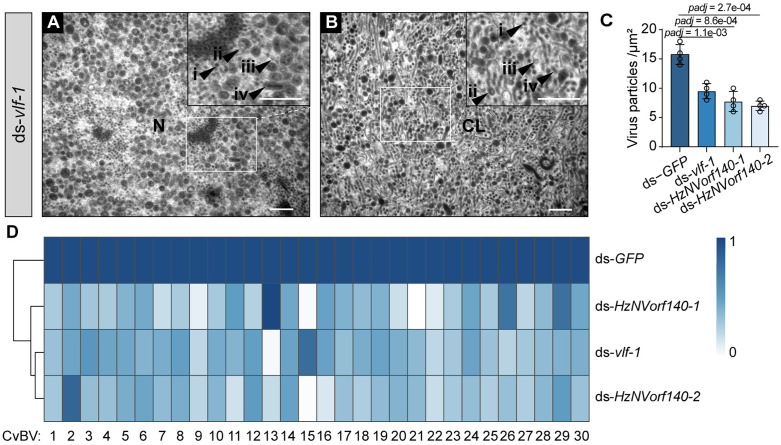
Knockdown of *vlf-1*, *HzNVorf140-1*, and *HzNVorf140-2* causes significant defects in CvBV virion assembly. **(A)** TEM image of calyx cell nuclei in FA1d female wasps pretreated with ds-*vlf-1*. Black arrows indicate four types of aberrantly assembled virions: **(i)** an unenveloped nucleocapsid in the nucleus; **(ii)** an envelope enclosing one or more incompletely processed, hollow nucleocapsids; **(iii)** an incompletely processed, hollow nucleocapsid lacking an envelope; and **(iv)** an envelope containing a nucleocapsid with an elongated, rod-like morphology. **(B)** TEM image of the calyx lumen in FA1d wasps pretreated with ds-*vlf-1*. Black arrows indicate the same aberrantly assembled virions described in **(A)**. **(C)** Virion density (number per μm²) in the calyx lumen of FA1d female wasps pretreated with ds-*GFP*, ds-*vlf-1*, ds-*HzNVorf140-1*, or ds-*HzNVorf140-2* (*n* = 4 biological replicates, measured from a 1 μm² central region selected in each of four independent images). The quantified virions included only particles containing both an envelope and a nucleocapsid, including most aberrantly assembled virions but excluding nucleocapsids lacking an envelope. *padj* values are shown. **(D)** Heatmap showing CvBV abundance in ovaries of FA1d female wasps pretreated with ds-*GFP*, ds-*vlf-1*, ds-*HzNVorf140-1*, or ds-*HzNVorf140-2* (*n* = 3 biological replicates). In the heatmap, the color of each cell represents the mean ratio of CvBV copy numbers from three biological replicates relative to the ds-*GFP* controls (raw CvBV copy numbers and corresponding raw and adjusted *p*-values are provided in S11 Table). The x-axis represents individual CvBV circles (circles 1–30). Lighter colors indicate a greater reduction in CvBV abundance following knockdown. N, nucleus; CL, calyx lumen. Scale bar: 0.5 μm. Statistical significance was assessed using two-sided *t*-tests with Benjamini–Hochberg correction.

Given that *vlf-1* is implicated in virion assembly of MdBV [27,28,33], we examined its function and that of its homologs *HzNVorf140–1* and *HzNVorf140–2* in *C. vestalis* [31]. RNAi knockdown of *vlf-1* caused four major defects in calyx cell nuclei: (i) an unenveloped nucleocapsid in the nucleus; (ii) an envelope enclosing one or more incompletely processed, hollow nucleocapsids; (iii) an incompletely processed, hollow nucleocapsid lacking an envelope; and (iv) an envelope containing a nucleocapsid with an elongated rod-like shape (Fig 5A, S8A, and S8C Fig). These defective virions were also released into the calyx lumen following calyx cell lysis and exhibited variable size (Fig 5B, S8B, and S8D Fig). Similarly, individual knockdown of the two *vlf-1* homologs (*HzNVorf140–1* and *HzNVorf140–2*) produced similar phenotypes in the calyx cell nuclei and calyx lumen. Based on TEM images of the calyx lumen, knockdown of each of the three genes caused a significant reduction in virion density, with remaining virions at comparable levels in all knockdowns (Fig 5C). Quantification of virion cross-sectional areas further showed that all three knockdowns significantly reduced virion size (S8E Fig). Similarly, qPCR analysis showed that knockdown of these three genes caused a significant overall reduction in CvBV abundance, without pronounced circle-specific effects (Fig 5D; raw CvBV copy numbers and *p*-values are shown in S11 Table). These findings indicate that *vlf-1* and its homologs are essential for CvBV virion assembly and maturation, with all three genes showing largely comparable effects.

### *Vp91* is essential for CvBV infectivity but dispensable for virion production

**Fig 6:**
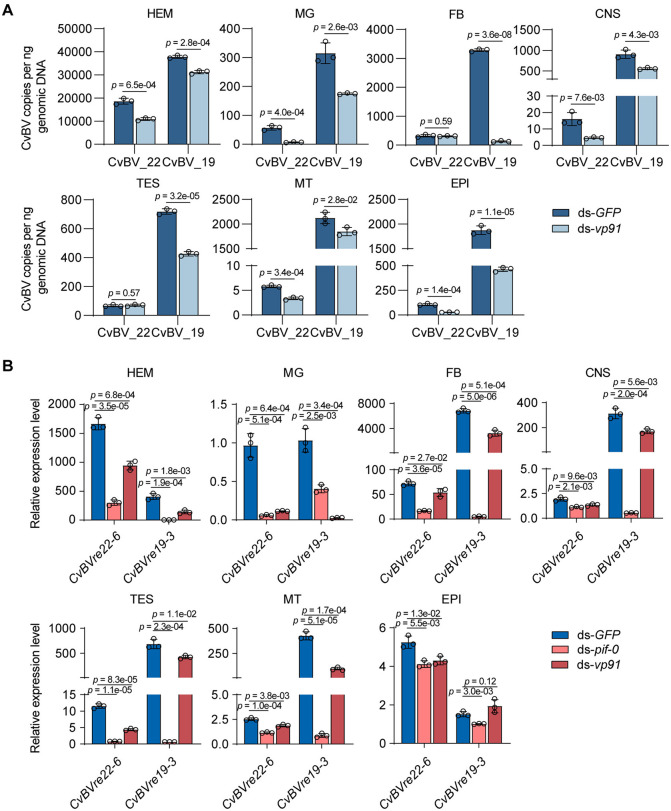
*vp91* functions as a viral infectivity factor. **(A)** Absolute quantification qPCR analysis of circular CvBV_19 and CvBV_22 copy numbers per ng of genomic DNA across seven *P. xylostella* tissues. Tissues were collected within 1 h after parasitization by one-day-old female wasps to minimize potential interference from viral genome integration into the host genome at later stages of parasitization. HEM, hemocytes; MG, midgut; FB, fat body; CNS, central nervous system; TES, testis; MT, Malpighian tubules; EPI, epidermis. *n* = 3 biological replicates. **(B)** qPCR analysis of the expression levels of two CvBV virulence genes in various tissues (same as in **(A)**) of *P. xylostella* at 24 h after parasitization by wasps pretreated with ds-*GFP*, ds-*pif-0*, or ds-*vp91* (*n* = 3 biological replicates). *p*-values, two-sided *t*-tests.

In calyx fluid samples, proteomic analysis identified several enriched proteins in addition to structural components and assembly-related factors. One of these proteins, *vp91*, is recognized as a per os infectivity factor (PIF-8) in AcMNPV [34–36], but its function in bracoviruses of *C. vestalis* remains unknown. In this study, we first examined the effect of *vp91* knockdown on virion morphology using TEM. We found that nucleocapsids and the surrounding envelope structures in calyx cell nuclei were largely unaffected, and the density of virions in the calyx lumen showed no obvious difference compared with ds-*GFP* controls (S9A-C Fig). Quantification of virion cross-sectional areas likewise revealed no obvious change in virion size following knockdown (S9D Fig). Moreover, qPCR analysis of CvBV circles 1–30 following *vp91* knockdown showed no significant changes in viral circle abundance (S9E Fig; raw CvBV copy numbers and *p*-values are shown in S12 Table), suggesting that *vp91* is unlikely to serve as a major structural component or assembly-related factor. To further evaluate whether *vp91* influences CvBV infection in the host, we quantified the absolute abundance of two CvBV circular DNAs, CvBV_19 and CvBV_22, in seven *P. xylostella* tissues within 1 h post-parasitization by ds-*vp91*-treated wasps, when most of the CvBV DNAs had not yet integrated into the host genome [31]. The abundance of both viral circles was reduced in most host tissues relative to the ds-*GFP* control group (Fig 6A), although CvBV_22 abundance in the fat body and testis showed no obvious decrease. Meanwhile, we detected CvBV gene expression in *P. xylostella* tissues parasitized by control or ds-*vp91*-treated wasps. Given that *pif-0* (*p74*) is a known per os infectivity factor in AcMNPV [27,37], we used knockdown of CvBV *pif-0* as a positive control to compare the effects of *vp91* knockdown. Knockdown of *vp91* markedly reduced the expression of the CvBV virulence genes *CvBVre22–6* and *CvBVre19–3* [38] across multiple *P. xylostella* tissues (Fig 6B). Notably, the extent of CvBV gene downregulation varied among different host tissues following *vp91* or *pif-0* knockdown. In the midgut, knockdown of *vp91* resulted in a marked reduction in CvBV expression, ranging from 8.5 to 38.8-fold, whereas in all other tissues, CvBV expression decreased more significantly following *pif-0* knockdown. These results indicate that *vp91* knockdown reduces CvBV accumulation across most host tissues. Consequently, the decreased expression of *CvBVre22–6* and *CvBVre19–3* is likely due to reduced viral infection efficiency and lower numbers of CvBV genomes delivered into host tissues. Together, these findings support a role for *vp91* as an infectivity factor required for efficient CvBV infection in host tissues, with no apparent role as a major structural component of CvBV virions.

## Discussion

Phylogenetic evidence suggests that BVs evolved from an ancestral nudivirus, and that both nudiviruses and baculoviruses descend from a more distant common ancestor [4,6,39]. Studies of AcMNPV and other baculoviruses have provided important insights into the functions of baculovirus core genes. Some homologs of these core genes identified in MdBV have been subjected to RNAi-mediated knockdown to assess their functions—representing the first functional analyses of nudivirus-like genes in BVs [27,28].

However, only a few nudivirus-like genes in BVs exhibit conserved functions comparable to their baculoviral homologs [1,3]. In *C. vestalis*, we aim to investigate whether these nudivirus-like genes play conserved roles in transcriptional regulation, virion structure and assembly, and infectivity. We further aim to determine whether their functions mirror or diverge from their counterparts in baculoviruses and MdBV.

Transcriptome analyses identified 71 nudivirus-like genes in *C. vestalis* (vs. 104 in *C. congregata* [22]), with expansion of the *odv-e66* family (*C. vestalis*: 20; *C. congregata*: 36). Given that *odv-e66* encodes a viral chondroitinase facilitating midgut infection in the baculovirus Bombyx mori nucleopolyhedrovirus (BmNPV) [40], this expansion may broaden the range of lepidopteran tissues that CvBV can infect. Our proteomic analysis of calyx fluid identified approximately 79% of nudivirus-like proteins in *C. vestalis*, providing a comprehensive overview of proteins incorporated into virions as well as those associated with viral functions but not packaged into virions. Using sucrose gradient density centrifugation, a multistep purification approach, we further defined 19 major nucleocapsid components enriched in purified virions. It should be noted that this purification strategy effectively enriches core structural proteins. However, it may result in the loss of viral proteins that are loosely associated with virions or only transiently interact with the particle surface. Consistent with this limitation, members of the PIF family and ODV-E66–like proteins were infrequently detected in purified virion fractions. This may reflect both their relatively low abundance, as reported for baculovirus PIF proteins [41], and the potential loss of loosely associated virion components during purification. Nevertheless, the proteins identified through this approach are expected to represent the principal structural components of CvBV virions.

While RNAi-based functional assays have inherent limitations, such as partial knockdown efficiency and off-target effects, it provides valuable insights into gene function. This is particularly true for endoparasitoid wasps, where the use of CRISPR-Cas9-based gene editing tends to be challenging, largely due to the considerable difficulties in embryo manipulation and rearing imposed by their parasitic lifestyle. Importantly, our RNAi-based functional validation demonstrated that these candidates appear to be important for nucleocapsid integrity, envelope formation, and virion assembly. This supports the specificity and overall purity of the proteomic dataset, even though it may not capture all virion-associated factors. In addition, several non-structural proteins, including the early-expressed proteins HELICASE, INTEGRASE-1, INTEGRASE-2, P47, LEF-4, LEF-5, LEF-8, and LEF-9, were detected only in calyx fluid. This observation further supports the relative purity of the purified virions.

Knockdown of *p47* and *lef-9* suggests that CvBV may encode a baculoviral-like RNA polymerase complex preferentially transcribing structural genes. Besides, *lef-5* knockdown reduced both early- and late-expressed gene expression and CvBV abundance, and TEM revealed severe virion assembly defects. In AcMNPV, *lef-5* functions as a late transcription factor without affecting early gene expression or DNA replication [33,42], suggesting that CvBV *lef-5* may have retained ancestral nudivirus functions while evolving new roles in endogenization. Consistently, comparative sequence and domain analyses show that CvBV LEF-5 differs substantially from its homologs in other baculoviruses and nudiviruses, including notable differences in predicted domain organization (S10 Fig), supporting the possibility of functional divergence. In addition, transcriptomic analyses identified eight gene expression clusters during ovary development, with *lef-5* located in cluster 2, which predominantly comprises wasp host genes. Although the potential cooperation between *lef-5* and these host gene clusters in regulating CvBV transcription and virion production is intriguing, it remains to be experimentally verified. Nevertheless, the precise molecular mechanisms underlying *lef-5* function remain unclear in both baculoviruses and bracoviruses. Future studies focusing on the molecular interactions and regulatory mechanisms of *lef-5* will be essential to elucidate its key roles in the transcriptional regulation of nudivirus-like genes.

Based on proteomics, 13 putative structural genes were selected for RNAi (capsid genes: *vp39*, *PmV*, *HzNVorf9–1*, *HzNVorf9–2*, *HzNVorf106*, *38k*, *27b*, and *K425_459*; envelope genes: *11k*, *17a-1*, *35a-1*, *35a-2*, and ds-*K425_461*). Knockdown of eight capsid genes disrupted CvBV morphology, producing empty envelopes or incompletely assembled virions, and globally reduced the abundance of CvBV circles 1–30. In addition, quantitative analysis of virion cross-sectional areas further suggested a broadly consistent reduction in virion size across capsid gene knockdowns, further supporting their conserved roles in virion morphogenesis. Among these, *vp39* knockdown caused the most severe defects, consistent with its role as a major capsid protein in baculoviruses [43,44]. In *M. demolitor*, *vp39*, *HzNVorf9–2*, *HzNVorf106*, and *27b* have also been identified as capsid genes, and knockdown of these genes produces broadly similar phenotypes [27,28]. In our study, *HzNVorf9–1*, a paralog of *HzNVorf9–2*, exhibited a phenotype similar to *HzNVorf9–2* and is therefore considered a capsid gene. However, in *M. demolitor*, *K425_459*, which lacks homologs in baculoviruses or nudiviruses, showed no visible effects on nucleocapsid morphology, virion morphogenesis, or proviral segment processing [28], suggesting functional diversification among bracovirus–wasp systems. The conserved *38k* gene, previously reported in AcMNPV to function in capsid assembly [44–46], is likewise essential for nucleocapsid integrity in *C. vestalis*, indicating functional conservation. Notably, we further identified *PmV*, a gene of nudivirus origin, as playing a critical role in maintaining CvBV nucleocapsid integrity.

Beyond capsid proteins, CvBV structural components include a class of envelope proteins that has remained largely uncharacterized in *C. vestalis* and is rarely verified in other braconids. In this study, we identified five envelope genes (*11k*, *17a-1*, *35a-1*, *35a-2*, and *K425_461*) based on qPCR-validated RNAi knockdown and TEM phenotypic analyses. Knockdown of these genes did not affect nucleocapsid formation but caused accumulation of unpackaged nucleocapsids in calyx nuclei, indicating defects in envelope formation. Notably, *11k* knockdown led to the accumulation of electron-dense nucleocapsid aggregates, suggesting it acts as a major envelope protein in CvBV and has functionally diverged from its role in AcMNPV, where it plays a role in primary oral infection [47]. We observed that knockdown of *35a-1* and *35a-2* did not affect nucleocapsid integrity or reduce virion abundance, a phenotype similar to that of the paralog *35a-5* in *M. demolitor* [28]. Notably, *35a-2* was previously detected in envelope-enriched proteomic fractions in *M. demolitor*, whereas *35a-5* was classified as a capsid protein [26], highlighting potential functional diversification within the *35a* gene family. In addition, we identified two other candidates, *17a-1* and *K425_461*, whose knockdown did not affect nucleocapsid formation but led to defects in envelope formation, suggesting that they also function as envelope components in *C. vestalis*. Interestingly, quantitative analysis of virion cross-sectional areas further suggested that knockdown of *17a-1* and *35a-1*/*35a-2* moderately reduced virion size, whereas no obvious size reduction was observed following *11k* or *K425_461* knockdown (S7N Fig). These observations suggest that different CvBV envelope-associated proteins may contribute unequally to virion morphogenesis and may possess partially distinct functional roles during envelope formation. Envelope proteins in large dsDNA viruses, including baculoviruses and bracoviruses, typically form multi-protein complexes embedded in a single virion membrane rather than existing independently for each capsid [19,48]. Such complexes allow multiple envelope components to cooperate during virion assembly and infection, and partial functional overlap among paralogous envelope genes is possible [41,48]. In our study, knockdown of the envelope genes (*17a-1*, *35a-1*, *35a-2*, and *K425_461*) led to relatively mild but broadly consistent phenotypes, suggesting that these genes may have partially redundant functions. While none of these genes appear to be major structural determinants of nucleocapsid assembly, their collective contributions to envelope formation and virion infectivity should be considered when interpreting single-gene knockdown results.

In *M. demolitor*, *vlf-1* knockdown disrupts virion assembly, produces defective virions, and impairs proviral DNA excision [27,28]. Phylogenetic analysis shows *vlf-1*, *HzNVorf140–1* (also named *vlf-1a*), and *HzNVorf140–2* (also named *vlf-1b*) form a paralogous clade [31], suggesting similar functions. Proteomics analyses indicate that these proteins are likely non-structural, consistent with their knockdown phenotypes: incompletely assembled virions are observed, and CvBV abundance is reduced—phenotypes reminiscent of those reported for AcMNPV *vlf-1* knockout mutants [49]. Upon knockdown of *vlf-1*, *HzNVorf140–1*, and *HzNVorf140–2*, CvBV abundance in the calyx lumen was significantly reduced, and unpackaged nucleocapsids were abundant, indicating that these genes are required for proper virion assembly and DNA packaging. Additionally, *HzNVorf140–2* (Gene ID: CVE05481) was previously reported to participate in CvBV integration [31]. Together, these results indicate that *vlf-1*, *HzNVorf140–1*, and *HzNVorf140–2* are likely required for DNA packaging and virion assembly, and may also act as integrases during replication and parasitism.

*Vp91*, a baculovirus core gene, was initially identified as a late-expressed gene in *Orgyia pseudotsugata* nucleopolyhedrovirus [50]. Its homolog, *p95*, in BmNPV is essential for BV production and nucleocapsid assembly [51], and *vp91* is recognized as a per os infectivity factor (PIF-8) in AcMNPV [34–36]. In CvBV, proteomics, qPCR, and TEM analyses indicate that *vp91* is not a structural protein and does not contribute to virion assembly. In contrast, tissue-specific absolute quantification of CvBV circular genomes demonstrates a reduction in viral accumulation across most host tissues upon *vp91* knockdown, together with decreased expression of viral virulence genes. These results suggest that *vp91* is required for efficient CvBV infection in host tissues by promoting viral delivery or infection success rather than virion assembly. Collectively, *vp91* appears to have undergone functional divergence in CvBV, being repurposed from a structural/assembly-associated role in baculoviruses to an infectivity-related factor in the braconid wasp-associated viral system.

**Fig 7:**
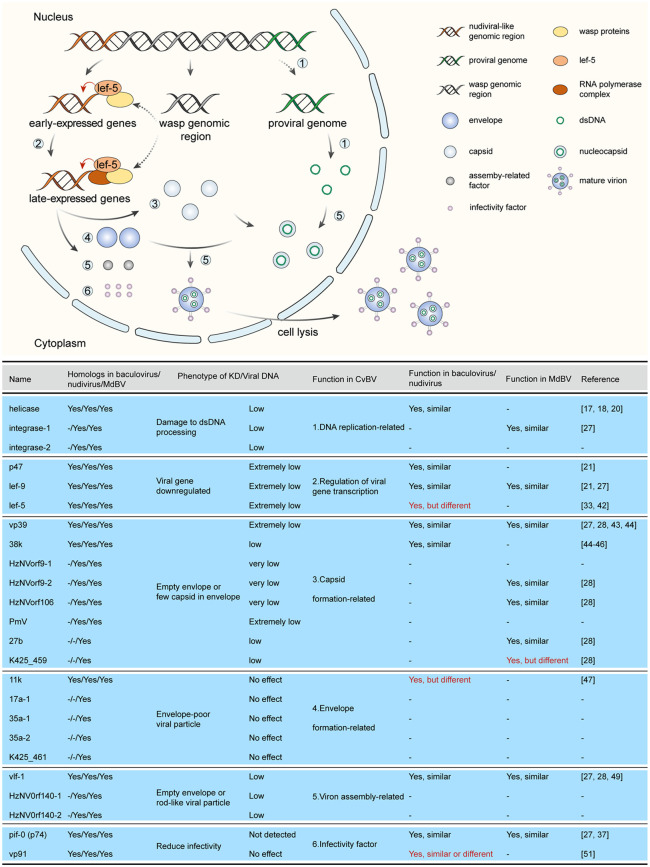
Multifunctional roles of nudivirus-like genes in the CvBV life cycle. The upper panel illustrates a schematic model showing the involvement of nudivirus-like genes in different stages of CvBV virion maturation. The parasitoid wasp genome comprises three components: (i) the nudivirus-like genomic region, (ii) the wasp genomic region, and (iii) the proviral genome, which harbors virulence genes. The early-expressed genes are primarily involved in the replication of CvBV dsDNA and the regulation of late-expressed gene expression. Whether parasitoid wasp genes are involved in this process remains unclear. The late-expressed genes mainly encode structural proteins as well as factors associated with virion assembly and host infection. Numbers 1–6 indicate the six functional categories of genes illustrated in the corresponding panels below. The lower panel lists the nudivirus-like genes identified in *C. vestalis* that play essential roles in CvBV production, morphogenesis, and infection. Knockdown of DNA replication-related genes likely interferes with proviral DNA segment processing, leading to a reduction in CvBV abundance; Knockdown of transcriptional regulatory genes reduces the expression of both early- and late-expressed genes; knockdown of capsid genes causes aberrant virion morphology, significantly reduces virion size, and a global reduction in CvBV abundance; knockdown of envelope genes impairs envelope formation, with some genes also causing reduced virion size, and slightly reduces CvBV abundance; knockdown of virion assembly-related genes severely disrupts CvBV virion assembly, resulting in multiple aberrantly shaped and significantly smaller virions, and markedly reduces CvBV abundance; and knockdown of *pif-0* or *vp91* severely impairs CvBV infectivity, although *vp91* knockdown does not affect virion size. In the table, the second column indicates the presence (“Yes”) or absence (“–”) of homologous genes in baculoviruses, nudiviruses, or MdBV. In columns 5 and 6, “Yes” indicates genes whose functions have been validated in baculoviruses, nudiviruses, or MdBV; red font highlights genes whose functions differ from those reported in baculoviruses, nudiviruses, or MdBV; “–” denotes genes whose orthologs have not been reported in baculoviruses, nudiviruses, or MdBV.

In summary, our integrative analyses—combining transcriptomics, proteomics, and RNAi-based functional assays—provide a comprehensive view of the functions of CvBV during virion production and parasitization. We identified a set of conserved structural proteins critical for nucleocapsid and envelope formation, along with several non-structural genes involved in DNA replication, transcriptional regulation, and capsid-envelope coordination (Fig 7). Comparative analyses with baculoviruses, nudiviruses, and MdBV revealed that while some nudivirus-like genes retain ancestral functions, others exhibit lineage-specific divergence, reflecting the adaptive specialization of CvBV within its wasp host. Collectively, our findings highlight both conserved and derived aspects of CvBV biogenesis, providing a valuable resource for studying bracovirus evolution and the functional innovation of symbiotic viruses.

## Materials and methods

### Insect rearing and sampling

*C. vestalis* and its host *P. xylostella* were maintained under standard laboratory conditions [52]. Developmental stages of *C. vestalis* were identified based on timing and morphology [32]. Ovaries were collected from females at four pupal stages (day 1: Ov1d, day 2: Ov2d, day 3: Ov3d, day 4: Ov4d) and one-day-old adults (OvFA1d), with three biological replicates per stage (30, 20, 6, 6, and 6 females, respectively) for RNA-seq.

### CvBV nudivirus-like genes identification

Bracovirus morphogenesis-related genes from *Cotesia congregata* were retrieved from published sources via ParWaspDB (https://bipaa.genouest.org/is/parwaspdb/) [22]. These served as queries for BLASTP searches against predicted coding sequences of the *C. vestalis* genome to identify putative CvBV nudivirus-like genes. De novo transcriptome assembly of all RNA-seq data was performed with Trinity v2.12.0 [53], and coding regions were predicted using TransDecoder v5.5.0. The resulting coding sequences were compared with the bracovirus dataset to refine CvBV gene identification. Genes within 10 kb were considered clusters.

### RNA-seq library construction, sequencing, and analyses

Total RNA from 15 ovary samples was used to construct RNA-seq libraries with Illumina kits (Illumina, USA) and sequenced as 150 bp paired-end reads on a HiSeq 2500 platform. Paired-end reads from *C. vestalis* ovary libraries were mapped to the reference genome (GenBank accession No. GCA_054085095.1) using HISAT2 v2.2.1 [54] with default settings. Gene-level read counts were generated from the mapped reads and used for differential expression analysis with DESeq2 [55]. Expression levels for visualization were quantified and normalized as transcripts per million (TPM). A combined protein dataset, including annotated genome proteins and newly predicted transcriptome proteins, was used for mass spectrometry-based annotation.

### Calyx fluid collection and CvBV virion purification

Two CvBV virion samples were isolated from *C. vestalis* ovaries for proteomic analysis: R1, representing calyx fluid, and R2, consisting of purified CvBV virions. The R2 sample was divided into two technical replicates (R2-1 and R2-2) to assess reproducibility for LC-MS/MS analysis (S3A Fig). Calyx fluid from 1,000 (R1) or 1,200 (R2-1 or R2-2) pairs of one- or two-day-old female wasps was collected and filtered through a 0.22 μm syringe filter (Merck Millipore, USA) to remove debris and eggs. The R1 filtrate was centrifuged (20,000 × g, 5 min, 4 °C) and washed three times with PBS to obtain a crude virion extract. The R2 sample underwent further purification by sucrose gradient centrifugation (25–65%, 100,000 × g, 2 h, 4 °C) as described previously [56]. The virion-containing fraction (45–55% sucrose) was collected, pelleted (20,814 × g, 2 h), and resuspended in ~400 μL PBS.

### Proteomic analysis of CvBV virions

Both calyx fluid (R1) and purified CvBV virions (R2) were used for mass spectrometry-based protein identification. In-gel trypsin digestion was performed overnight at 37 °C with 3 µg trypsin (Promega, USA) in 100 mM NH_4_HCO_3_. Peptides were desalted using C18 cartridges (Sigma-Aldrich, USA), vacuum-dried, and reconstituted in 0.1% formic acid. LC-MS/MS analysis was conducted on an LTQ Orbitrap Elite mass spectrometer coupled to an Easy-nLC II system (Thermo Fisher Scientific, USA). Peptides were loaded onto a trap column (75 µm × 2 cm, C18) and separated on an analytical column (50 µm × 15 cm, C18) using a five-step linear gradient from 3% to 90% acetonitrile (0.1% formic acid) over 150 min at 250 nL/min. Data were acquired in data-dependent mode with the top 20 ions selected for CID MS/MS. Proteins were identified by searching against a custom database of predicted ORFs from the ovary transcriptome datasets, and only proteins supported by at least two unique peptide spectrum matches were retained.

### Gene cloning and verification

RT-PCR was performed with KOD polymerase (TOYOBO, Japan) to validate full-length or partial ORFs of CvBV nudivirus-like genes. For selected genes, mRNA expression was validated by qPCR using total RNA extracted from pooled ovaries (6–20 per sample) collected at four developmental stages (Ov2d, Ov3d, Ov4d, and OvFA1d). qPCR primers listed in S13 Table. Ribosomal protein *Cv_18s rRNA* (GenBank JX399880.1) and *Cv_*β-*tubulin* (GenBank MT459787) were used as reference genes for *C. vestalis*, while *Px_*β-*tubulin* (GenBank EU127912) and *Px_*β-*actin* (GenBank NM_001309101) served as internal controls for *P. xylostella*. We used the comparative 2^−ΔΔCT^ method to calculate the relative gene expression levels [57].

### RNAi of selected genes

Gene-specific primers with T7 promoter adaptors (S13 Table) were used to amplify 300–900 bp templates for dsRNA synthesis. dsRNA was synthesized using the MegaScript RNAi Kit (Ambion, USA). A 0.1 μL dsRNA solution (2–5 μg/μL) was injected into one-day-old female pupae using a Fentojet Express microinjector (Eppendorf, Germany) and a Narishige micromanipulator, as previously optimized for RNAi efficiency [58]. Controls were injected with non-specific *GFP* dsRNA. One-day-old adults were sampled, and ovaries were dissected with ophthalmic scissors for downstream analysis. In addition, off-target siRNA generation was evaluated using DSIR (http://biodev.extra.cea.fr/DSIR/DSIR.html) predictions. Importantly, only homologous regions overlapping dsRNA target sequences were considered potential off-target sites, whereas matches outside target regions were excluded. Primer information for all off-target analyses is provided in S13 Table.

### Absolute quantification of CvBV circles by qPCR

To quantify 30 CvBV circles, genomic DNA was extracted from the ovaries of pupae and adult females using the Wizard Genomic DNA Purification Kit (Promega, USA) following the manufacturer’s instructions. Primers and absolute quantification curves are described in [31]. For absolute quantification of CvBV abundance in different *P. xylostella* tissues, genomic DNA was isolated from hemocytes, midgut, fat body, central nervous system, testis, Malpighian tubules, and epidermis collected within 1 h after parasitization by one-day-old female wasps to minimize potential interference from viral genome integration at later stages of parasitization. The copy numbers of circular CvBV_19 and CvBV_22 were quantified by absolute qPCR and normalized to total genomic DNA input, and the abundance of each viral circle was calculated as copy number per ng of genomic DNA.

### TEM observation

Ovaries from one- or two-day-old female wasps were dissected and fixed in 2.5% glutaraldehyde in PBS at 4 °C for 8–12 h. Non-essential ovarian tubules were removed, and calyx regions containing CvBV particles were post-fixed in 1% osmium tetroxide at room temperature for 1 h. Samples were dehydrated through graded ethanol, embedded in resin, and ultrathin sections were cut with a LEICA EM UC7 microtome. Sections were double-stained with lead citrate and Uranyless (DeltaMicroscopy, France) for 5–10 min and examined on a Hitachi H7650 electron microscope at 100 kV (Bio-ultrastructure Analysis Lab, Zhejiang University). At least three females were analyzed per dsRNA treatment to ensure consistent observations.

### Statistics

Graphs and statistical analyses were performed using GraphPad Prism 8 (GraphPad, USA). Two-sided Student’s *t*-tests were used to assess differences between groups. Multiple-testing correction using the Benjamini–Hochberg method was applied when multiple hypotheses were tested within the same experimental framework. Adjusted *p-*values < 0.05 were considered statistically significant. Exact *n*, *p*-values and adjusted *p*-values are provided in figure legends. Data are presented as mean ± s.d. unless stated otherwise. Heatmaps were generated with pheatmap v1.0.12 in R v4.1.0.

## Supporting information

S1 Fig Ovary and calyx cell nuclear morphology during pupal development in *C. vestalis.* **(A–I)** Ovary morphology of female pupae and adult at different stages: 1-day-old (Ov1d), 1.5-day-old (Ov1.5d), 2-day-old (Ov2d), 2.5-day-old (Ov2.5d), 3-day-old (Ov3d), 3.5-day-old (Ov3.5d), 4-day-old (Ov4d), 4.5-day-old (Ov4.5d), and 1-day-old female adult (OvFA1d). During ovarian development, calyx cells proliferate rapidly, gradually occupying the entire calyx lumen as wasp eggs mature. The yellow regions shown in **(H)** and **(I)** indicate mature wasp eggs. At OvFA1d, the distinct locations of the calyx region, wasp eggs, and ovarioles are illustrated. **(J–L)** DAPI-stained calyx cell nuclei at Ov3d **(J)**, Ov4d **(K)**, and OvFA1d **(L)**. From Ov3d to OvFA1d, calyx cells producing CvBV virions exhibit enlarged nuclei. **(M)** Magnified view of the VS shown in Fig 1D, and black arrows indicate nucleocapsids lacking an envelope, accumulating in the VS region awaiting assembly. **(N)** After the calyx cells' lysis, the calyx fluid, containing mature virions, surrounds the wasp egg in the calyx lumen, preparing it for injection into the host. **(O)** Magnified view of the white box in **(N)**, showing numerous mature virions in contact with a wasp egg. N, nucleus. VS, virogenic stroma. CL, calyx lumen. E, wasp egg. Scale bars: **(A-L)**, 0.1 μm; **(M)**, 0.2 μm; **(N)**, 5 μm; **(O)**, 0.5 μm.
(TIF)

S2 Fig Differential gene expression in *C. vestalis* ovaries across pupal to adult developmental stages. **(A)** K-means clustering (*k* = 8) of gene expression profiles across five ovarian developmental stages. Solid lines indicate average expression and shaded areas show standard deviation for each cluster. **(B)** Chromosomal localization of 71 CvBV nudivirus-like genes, which can be divided into seven clusters. Red arrowheads indicate 5’–3’ orientation; blue arrowheads indicate 3’–5’. Genes within 10 kb of each other were grouped into the same cluster.
(TIF)

S3 Fig Correlation analysis of mass spectrometry and transcriptome data. **(A)** Workflow for mass spectrometry analysis of crude virion extracts (R1) and purified virions (two technical replicates: R2-1 and R2-2). **(B)** Mass spectrometry replicates are highly reproducible (*r* > 0.6) and correlate strongly with transcriptome data (*r* > 0.49), indicating consistent multi-omics profiles. The PSMs and unique peptide counts were directly used for correlation analysis. For genes supported by ≥ 2 unique peptides, their log_2_-transformed FPKM values at Ov4d were correlated with both PSM and unique peptide counts.
(TIF)

S4 Fig Functional validation of nudivirus-like genes via RNAi. **(A)** Schematic showing the RNAi experimental workflow. **(B)** qPCR analysis showed high knockdown efficiency across different genes (*n* = 3 biological replicates). **(C)** Screenshot summarizing pairwise nucleotide homology analysis among CvBV nudivirus-like genes. For each comparison, the overlap between the query region and the dsRNA target region is shown. Self-alignments were excluded, and alignments ≥ 21 nt between different genes were considered potential off-target regions. **(D and E)** qPCR analysis showed efficient knockdown of *HzNVorf9–1* or *HzNVorf106* transcripts in FA1d female wasps pretreated with ds-*HzNVorf9–1* **(D)** or *ds-HzNVorf106* **(E)**, respectively (*n* = 3 biological replicates). Potential off-target genes were also examined and showed no significant changes in expression. *p*-values, two-sided *t*-tests.
(TIF)

S5 Fig Capsid gene knockdown causes severe morphological defects in CvBV virions. **(A–R)** TEM images of virion morphogenesis in FA1d female wasps following RNAi knockdown of capsid genes. **(A**, **D**, **G**, **J**, **M and P)** Overview of the calyx cell nuclei in FA1d female wasps pretreated with ds-*vp39*, ds-*HzNVorf9–1*, ds-*HzNVorf9–2*, ds-*HzNVorf106*, ds-*27b* or ds-*K425_459*. **(B, E, H, K, N and Q)** Magnified views of boxed areas in **(A, D, G, J, M and P)**, respectively. The insets (black boxes) show magnified views of the regions outlined by white boxes, highlighting the effects of RNAi on virion assembly. **(B, E, H and K)** Black arrows indicate two types of aberrantly assembled virions: **(i)** an empty envelope lacking nucleocapsids (a large hollow circle), which represents the most frequently observed phenotype; and **(ii)** a single large, irregularly shaped nucleocapsid enclosed by an envelope (a large hollow circle containing a slightly smaller hollow circle). **(N and Q)** Black arrows indicate three types of aberrantly assembled virions: **(i)** a complete nucleocapsid lacking an envelope (a small electron-dense dot); **(ii)** an empty envelope lacking nucleocapsids (a large hollow circle); and **(iii)** a partially assembled virion containing only a nucleocapsid enclosed by a single-membrane envelope (an electron-dense dot within a membrane-bound circle). **(C, F, I, L, O, and R)** In contrast to ds-*GFP* controls, mature virions are largely absent from the calyx lumen. N, nucleus; E, wasp egg; CL, calyx lumen. Scale bars: **(A, D, G, J, M, and P)**, 5 μm; **(B, C, E, F, H, I, K, L, N, O, Q, and R)**, 0.5 μm; insets of **(B, E, H, K, N, and Q)**, 0.25 μm.
(TIF)

S6 Fig Quantification of virion cross-sectional area in the calyx lumen following knockdown of capsid genes. Cross-sectional area (μm²) of individual virions in the calyx lumen of FA1d female wasps pretreated with ds-*GFP*, ds-*vp39*, ds-*PmV*, ds-*HzNVorf9–1*, ds-*HzNVorf9–2*, ds*-HzNVorf106*, ds-*38k*, *ds-27b*, or ds*-K425_459*, as quantified from TEM images. A total of *n* = 50 randomly selected, non-overlapping virions without obvious aggregation were measured in each group. Median values are indicated on the plot. N, nucleus; E, wasp egg; *padj* values are shown. Statistical significance was assessed using two-sided *t*-tests with Benjamini–Hochberg correction.
(TIF)

S7 Fig Envelope gene knockdown has a minor effect on CvBV abundance and impairs virion integrity. **(A–L)** TEM images of virion morphogenesis in FA1d female wasps following RNAi knockdown of envelope genes. **(A, D, G, and J)** Overview of the calyx cell nuclei in FA1d female wasps pretreated with ds-*17a-1*, ds-*35a-1*, ds-*35a-2*, or ds-*K425_461*. **(B, E, H, and K)** Magnified views of the boxed areas in **(A, D, G, and J)**, respectively. White dashed circles highlight regions where multiple nucleocapsids, lacking an envelope, are aggregated together. **(C, F, I, and L)** TEM images of the calyx lumen in FA1d female wasps pretreated with ds-*17a-1*, ds-*35a-1*, ds-*35a-2*, or ds-*K425_461.* White arrows indicate nucleocapsids not enclosed by an envelope that are released into the calyx lumen following cell lysis. **(M)** Virion density (number per μm²) in the calyx lumen of FA1d female wasps pretreated with ds-*GFP*, ds-*11k*, ds-*17a-1*, ds-*35a-1*, ds-*35a-2*, or ds-*K425_461,* as quantified from TEM images (*n* = 4 biological replicates, measured from a 1 μm² central region selected in each of four independent images). **(N)** Cross-sectional area (μm²) of individual virions in the calyx lumen of FA1d female wasps pretreated with ds-*GFP*, ds-*11k*, ds-*17a-1*, ds-*35a-1*, ds-*35a-2*, or ds-*K425_461,* as quantified from TEM images. A total of *n* = 50 randomly selected, non-overlapping virions without obvious aggregation were measured in each group. Median values are indicated on the plot. *padj* values are shown. **(O)** Heatmap showing CvBV abundance in ovaries of FA1d female wasps pretreated with six dsRNAs shown in **(M)** (*n* = 3 biological replicates). In the heatmap, the color of each cell represents the mean ratio of CvBV copy numbers from three biological replicates relative to the ds-*GFP* controls (raw CvBV copy numbers and corresponding raw and adjusted *p*-values are provided in S10 Table). The x-axis represents individual CvBV circles (circles 1–30). Lighter and darker colors indicate slight decreases and increases in CvBV abundance, respectively, following knockdown. N, nucleus; CL, calyx lumen. Scale bars: **(A, D, G, and J)**, 5 μm; **(B, C, E, F, H, I, K, and L)**, 0.5 μm. Statistical significance was assessed using two-sided *t*-tests with Benjamini–Hochberg correction. ns, not significant.
(TIF)

S8 Fig Knockdown of *HzNVorf140–1* and *HzNVorf140–2* causes significant defects in CvBV virion assembly. **(A–D)** TEM images of calyx cell nuclei **(A and C)** and calyx lumen **(B and D)** in FA1d wasps pretreated with ds-*HzNVorf140–1* **(A and B)** or ds-*HzNVorf140–2* **(C and D)**. Black arrows indicate four types of aberrantly assembled virions, as described in Fig 5A: **(i)** an unenveloped nucleocapsid in the nucleus; **(ii)** an envelope enclosing one or more incompletely processed, hollow nucleocapsids; **(iii)** an incompletely processed, hollow nucleocapsid lacking an envelope; and **(iv)** an envelope containing a nucleocapsid with an elongated, rod-like morphology. **(E)** Cross-sectional area (μm²) of individual virions in the calyx lumen of FA1d female wasps pretreated with ds-*GFP*, ds-*vlf-1*, ds-*HzNVorf140–1*, or ds-*HzNVorf140–2,* as quantified from TEM images. A total of *n* = 50 randomly selected, non-overlapping virions without obvious aggregation were measured in each group. Median values are indicated on the plot. VS, virogenic stroma; N, nucleus; CL, calyx lumen. Scale bar: 0.5 μm. *padj* values are shown. Statistical significance was assessed using two-sided *t*-tests with Benjamini–Hochberg correction.
(TIF)

S9 Fig *vp91* functions as a viral infectivity factor. **(A)** TEM image of calyx cell nuclei in FA1d female wasps pretreated with ds-*vp91*. **(B)** TEM image of calyx lumen in FA1d female wasps pretreated with ds-*vp91*. **(C)** Virion density (number per μm²) in the calyx lumen of FA1d female wasps pretreated with ds-*GFP* or ds-*vp91* (*n* = 4 biological replicates, measured from a 1 μm² central region selected in each of four independent images). *p*-values, two-sided *t*-tests. **(D)** Cross-sectional area (μm²) of individual virions in the calyx lumen of FA1d female wasps pretreated with ds-*GFP* or ds-*vp91,* as quantified from TEM images. A total of *n* = 50 randomly selected, non-overlapping virions without obvious aggregation were measured in each group. Median values are indicated on the plot. *p*-values, two-sided *t*-tests. **(E)** qPCR analysis showing the relative abundance of CvBV circles 1–30 in ds-*vp91*-injected wasps, normalized to ds-*GFP* controls as defined in Fig 3A (*n* = 3 biological replicates; raw CvBV copy numbers and corresponding raw and adjusted *p*-values are provided in S12 Table). N, nucleus; CL, calyx lumen. Scale bars: **(A)**, 5 μm; **(B)**, 0.5 μm. Statistical significance was assessed using two-sided *t*-tests with Benjamini–Hochberg correction. ns, not significant.
(TIF)

S10 Fig Amino acid sequence alignment of CvBV LEF-5 with homologs from related bracoviruses, baculoviruses, and nudiviruses. The alignment was generated using MEGA12 and visualized with GeneDoc v2.7.0. Percent amino acid similarity between CvBV LEF-5 and each homolog is indicated in parentheses next to the gene names. Conserved residues are indicated by color: red, highly conserved; blue, moderately conserved; gray, weakly conserved. Red boxes indicate the predicted Baculo_LEF5_C domain, which shows substantial sequence variation across species. This C-terminal domain is predicted to function as a zinc-binding domain and may contribute to the functional divergence of CvBV LEF-5 relative to its homologs. CvBV, Cotesia vestalis bracovirus (sequence in S3 Table); CcBV, Cotesia congregata bracovirus (CAD6209794.1); MdBV, Microplitis demolitor bracovirus (NP_001401715.1); AcMNPV, Autographa californica multiple nucleopolyhedrovirus (AAA66729.1); HzNV-2, Heliothis zea nudivirus 2 (AEW69589.1); OrNV, Oryctes rhinoceros nudivirus (ABF93324.1).
(TIF)

S1 Table Copies of CvBV circles 1–30 per individual ovary during pupal developmental stages (*n* = 3 biological replicates). (XLSX)

S2 Table RNA-seq data summary. (XLSX)

S3 Table The 71 conserved nudivirus-like genes identified in the *C. vestalis* genome. (XLSX)

S4 Table Raw read counts, TPM values, and DESeq2 differential expression analysis of *C. vestalis* genes, including 71 nudivirus-like genes, in ovaries across different developmental stages. (XLSX)

S5 Table Protein abundances identified in calyx fluid by mass spectrometry analysis (unique peptides ≥ 2 are shown). (XLSX)

S6 Table Protein abundances identified in purified CvBV virions by mass spectrometry analysis (unique peptides ≥ 2 are shown). (XLSX)

S7 Table Relative abundance of CvBV circles 1–30 in FA1d female wasps pretreated with ds-*GFP*, ds-*helicase*, ds-*integrase-1*, or ds-*integrase-2* (*n* = 3 biological replicates). (XLSX)

S8 Table Relative abundance of CvBV circles 1–30 in FA1d female wasps pretreated with ds-*GFP*, ds-*p47*, ds-*lef-5*, or ds-*lef-9* (*n* = 3 biological replicates). (XLSX)

S9 Table Relative abundance of CvBV circles 1–30 in FA1d female wasps pretreated with ds-*GFP*, ds-*vp39*, ds-*PmV*, ds-*HzNVorf9–1*, ds-*HzNVorf9–2*, ds*-HzNVorf106*, ds-*38k*, *ds-27b*, or ds*-K425_459* (*n* = 3 biological replicates). (XLSX)

S10 Table Relative abundance of CvBV circles 1–30 in FA1d female wasps pretreated with ds-*GFP*, ds-*11k*, ds-*17a-1*, ds-*35a-1*, ds-*35a-2*, or ds-*K425_461* (*n* = 3 biological replicates). (XLSX)

S11 Table Relative abundance of CvBV circles 1–30 in FA1d female wasps pretreated with ds-*GFP*, ds-*vlf-1*, ds-*HzNVorf140–1*, or ds-*HzNVorf140–2* (*n* = 3 biological replicates). (XLSX)

S12 Table Relative abundance of CvBV circles 1–30 in FA1d female wasps pretreated with ds-*GFP* or ds-*vp91* (*n* = 3 biological replicates). (XLSX)

S13 Table Primers used for PCR cloning, dsRNA synthesis, and qPCR. (XLSX)
